# High content, high-throughput screening for small molecule inducers of NF-κB translocation

**DOI:** 10.1371/journal.pone.0199966

**Published:** 2018-06-28

**Authors:** Samuel Njikan, Alyssa J. Manning, Yulia Ovechkina, Divya Awasthi, Tanya Parish

**Affiliations:** TB Discovery Research, Infectious Disease Research Institute, Seattle, WA, United States of America; University of South Alabama Mitchell Cancer Institute, UNITED STATES

## Abstract

NF-κB is an important mediator of immune activity and its activation is essential in mounting immune response to pathogens. Here, we describe the optimization and implementation of a high-throughput screening platform that utilizes high content imaging and analysis to monitor NF-κB nuclear translocation. We screened 38,991 compounds from three different small molecule libraries and identified 103 compound as hits; 31% of these were active in a dose response assay. Several of the molecules lacked cytotoxicity or had a selectivity index of more than 2-fold. Our image-based approach provides an important first step towards identifying small molecules with immunomodulatory activity.

## Introduction

Nuclear factor kappa B (NF-κB) is a protein complex found in almost all eukaryotic and it is involved in the expression and regulation of a wide range of genes involved in cellular response to stimuli, transcription of DNA, cell survival and proliferation as well as cytokine production [1–3]. It is mostly found as a p65 homodimer or p65/p50 heterodimer with the p65 component responsible for most of its transcription factor function [4]. With the exception of keratinocytes and vascular smooth muscles, it is predominantly found in the cytoplasm of all cells bound to the inhibitory molecule IκB ([5, 6]. The size of this complex together with blocking of the nuclear localization signals by IκB prevents NF-κB from translocating to the nucleus. Upon exposure to external stimuli, a series of signaling cascades are triggered which result in the degradation of IκB by phosphorylation leading to NF-κB activation and its translocation into the nucleus [7]. Following NF-κB activation and translocation, IκB accumulates in the cytoplasm [8, 9], is translocated to the nucleus and binds to NF-κB inhibiting its activity completing the cycle ([10].

Substances or agents capable of inducing NF-κB nuclear translocation and activation are promising vaccine adjuvants [11]. Several mechanisms have been described linking NF-κB activation to adjuvant activity, including toll like receptors (TLRs) signaling and kinase activity [12, 13]. TLRs are found largely on immune cells [14, 15], but are also expressed on other cell types including endothelial cells [16]. By exerting their effects in boosting the immune response, adjuvants have the potential to be used in adjunctive therapy to shorten treatment regimen as well as facilitate clearance of pathogens in a host-directed manner [17–19]

Quantitative measurement of NF-κB nuclear translocation is a critical research tool for early drug discovery as well as cellular immunology. Several methods have been used to measure NF-κB in the nucleus [20–23]. However, these methods are limited by their sensitivity and laborious procedures. Recently, novel imaging methods using confocal microscopy [4] or automated fluorescent microscopy with computer assisted image analysis technology [24] have been described to detect NF-κB nuclear translocation. The latter, known as High Content Imaging (HCI), is based on the phenotypic assessment of several biological properties. It not only offers the high-throughput platform but also provides the advantage of measuring multiple cellular characteristics such as such as cell cycle, motility, morphology, receptor internalization and protein redistribution. This platform has been widely applied in screening large compound libraries in drug discovery as well as cellular immunology to identify and/or understand novel drug targets [25, 26].

In this study, we used a high content imaging platform to screen for compounds which induce the nuclear translocation of NF-κB. The assay was developed for high-throughput screening with endothelial cells but can be adapted for low-throughput and other cell types with some modifications.

## Materials and methods

### Maintenance of cell lines

Human umbilical vein endothelial cells (HUVECs, Lonza, USA) were maintained at 37°C in a humidified 5% CO_2_ incubator in complete endothelial cell growth medium (EGM^TM^-2 BulletKit^TM^ Medium) consisting of Endothelial Basal Medium with SingleQuot supplement (epidermal growth factor, vascular endothelial growth factor, R3- insulin-like growth Factor-1, ascorbic acid, hydrocortisone, human fibroblast growth factor-beta, heparin, gentamicin/amphotericin-B) plus 2% fetal bovine serum (FBS).

The HepG2 human cell line (ATCC HB-8065) was propagated in Dulbecco’s Modified Eagle Medium (DMEM) containing either 25 mM D-glucose or 10 mM D-galactose [27], 10% FBS, 1 mM sodium pyruvate, 2 mM Glutagro (Corning), and 100 I.U/mL penicillin and 100 μg/mL streptomycin.

### Preparation of chemical library

Compound plates were prepared in 384-well plates for screening. Compounds stocks (1mM in DMSO) were dispensed into columns 3–22 in DMSO and tested at a final concentration of 10 μM (1% final DMSO). All compounds were supplied at >90% purity as per vendor.

### NF-κB nuclear translocation assay

HUVEC cells were seeded at 2500 cells/well in 384-well plates in EBM^TM^-2 Basal Medium plus 2% FBS overnight at 37°C in a humidified 5% CO2 incubator. Compounds (columns 3–22) and DMSO (columns 1, 2, 23 and 24) were added to cells using a JANUS Automated Liquid Handling Workstation (Perkin Elmer). Tumor Necrosis Factor-alpha (TNF-α) recombinant protein (eBiosciences) was used as positive control and was added to columns 1 and 24 at a final assay concentration of 100 ng/ml while DMSO served as negative control. Plates were incubated at 37°C for 30 min in a humidified 5% CO_2_ incubator following addition of compounds and controls.

All washing and immunofluorescence staining steps were performed using an EL406 Automated Washer Dispenser (Biotek). Briefly, cells were fixed with 4% paraformaldehyde at RT for 15 min. Cells were washed and incubated at RT for 30 min in 0.05% Triton X-100, 0.25% IgG-free BSA, 0.25% normal goat serum in Tris-buffered saline. Cells were stained with the p65 Mouse Monoclonal Primary Antibody (Santa Cruz, sc-8008) at a final concentration of 1 μg/mL in TBS, 0.05% Triton X-100,.25% IgG-free BSA and incubated overnight at 4°C. Cells were washed and stained with the Alexa Fluor 488 Goat anti-Mouse IgG (H+L) cross-adsorbed secondary antibody at a final concentration of 4 μg/mL in TBS, 0.05% TritonX-100, 0.25% IgG-free BSA for 1 hr at RT in the dark. Cells were washed and 1 μg/mL of DAPI added. Plates were sealed and imaged with an ImageXpress Micro XLS High Content Screening System (Molecular Devices) using FITC and DAPI channels at 20X magnification. Nine fields were captured for each well.

### Image analysis

Images were analyzed using the MetaXpress software (Molecular Devices) by creating a custom analysis program in the Custom Module Editor. Briefly, DAPI was used to identify the nucleus of the cell and a nuclear mask created. A secondary mask was created by enlarging the nucleus mask to identify the whole cell. The whole cell mask was then overlaid on the nucleus mask to identify the cytoplasm. The average p65 (NF-κB) pixel intensities in both the nucleus and cytoplasm were quantified and a value assigned to each. A translocation value was calculated by determining the ratio of the average nuclei intensity to the average cytoplasmic intensity of p65 (NUC/CYT) [24]. For assay development, the Kruskal-Wallis non parametric test was used to compare the medians of the different groups after correcting for multiple comparison with Dunn’s multiple comparison test using GraphPad Prism 7.04 software. For assay validation, the mean (μ), standard deviation (SD) and coefficient of variation (CV) for nuclear translocation were calculated for the maximum (TNF-α) and minimum (DMSO) activation control wells, maximum and minimum activation plates as well as CVs for 320 test wells. The Z’ (measure of spread between maximum and minimum control signals) was calculated for each plate using the formula:

$$Z^{\prime }=1-\frac{3({SD}_{Max}+{SD}_{Min})}{\left|\mu_{Max}-\mu_{Min}\right|},$$

where max is average maximum signal and min is average minimum signal [28]. The EC_50_ was defined as the concentration that gave half the maximal response.

### HepG2 cytotoxicity assay

Cells were seeded in 384-well plates at 1800 cells per well and incubated in a humidified incubator at 37°C, 5% CO2. Compounds were solubilized in 100% DMSO and assayed using a 10-point three-fold serial dilution. Compounds were added 24 hours post cell seeding to a final assay concentration of 1% DMSO and highest compound concentration of 100 μM. CellTiter-Glo® reagent (Promega) was added to 384-well plates after a 72-hour incubation period. Relative luminescent units (RLU) were measured using a Synergy 4 plate reader (Biotek). Raw data were normalized using the average RLU value from negative control (1% DMSO) and expressed as % growth. Growth inhibition curves were fitted using the Levenberg–Marquardt algorithm. The IC_50_ was defined as the compound concentration that produced 50% of the growth inhibitory response.

## Results

### Assay development

**Fig 1:**
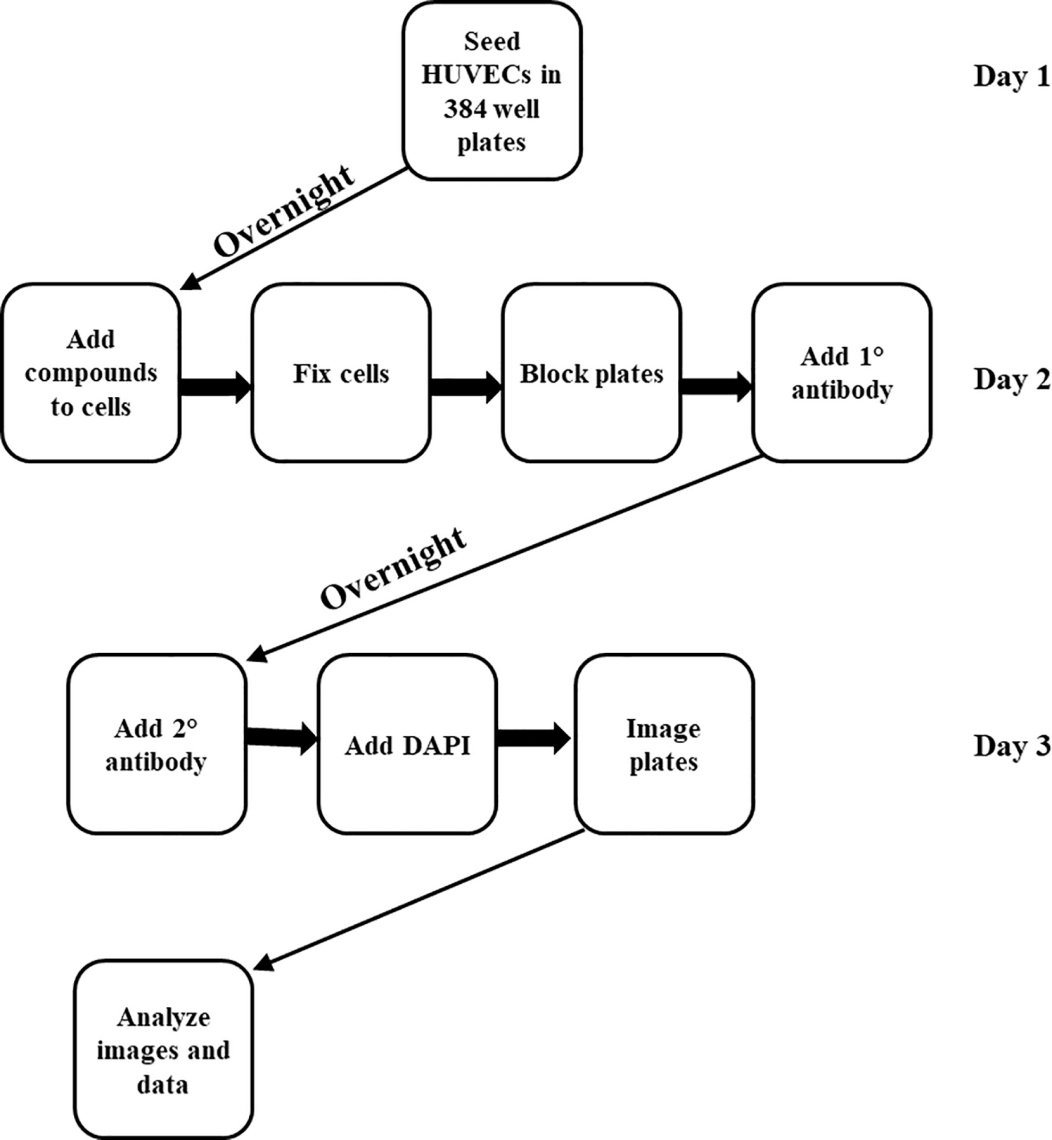
Schematic representation of NF-kB nuclear translocation assay.

In this study, we aimed to identify compounds that induced NF-κB nuclear translocation by high content imaging using HUVECs. We optimized and validated a screen to use high-throughput microscopy on 384-well plates. Our screen was developed using an overall workflow (Fig 1) in order to allow us to optimize key parameters. In overview, HUVECs were seeded into 384-well plates and incubated in a humidified incubator at 37°C, 5% CO_2_ overnight. Compounds and controls were added to plates and cells incubated for a further time. Cells were fixed with paraformaldehyde, washed, blocked and incubated with the NF-κB primary antibody overnight. An Alexa Fluor conjugated secondary antibody was used to stain NF-κB and DAPI was used to stain cell nuclei. Plates were imaged in both FITC and DAPI channels using the ImageXpress high content microscope.

We optimized a number of parameters. We first determined the optimum cell seeding density. For accurate measurement of NF-κB, it was important to identify individual cells as well as cellular segmentation in a monolayer of adherent cells after overnight seeding. We tested four different seeding densities (8000, 4000, 2000 and 1000 cells/well) and analyzed cell-to-cell contact after overnight incubation (data not shown). We observed adequate contact between cells with no piling and clumping with 2000 cells. Higher cell densities (4000 and 8000 cells/well) resulted in piling and clumping of cells, while wells seeded with 1000 cells had little or no cell contact between the cells. We chose 2500 cells/well as seeding density in order compensate for any cell loss during the washing steps.

**Fig 2:**
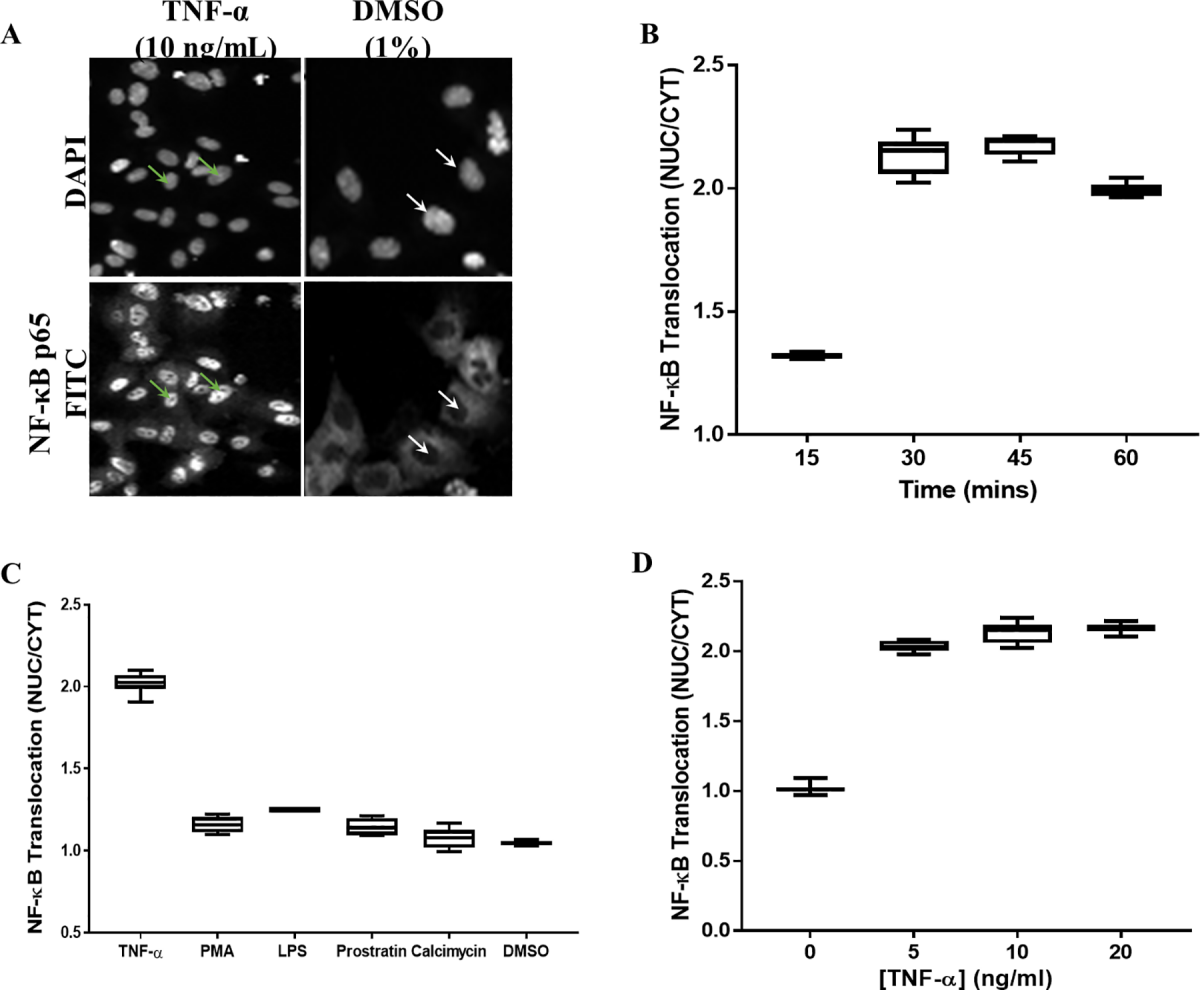
Optimizing activation stimuli for NF-κB nuclear translocation. HUVECs were seeded overnight, stimulated with TNF-α for 30 min and stained for NF-κB. (A) Images from the DAPI and FITC channel. White arrows indicate non-stained nuclei in FITC channel; green arrows showing stained nuclei in FITC channel (B) Time course of translocation. HUVECs were stimulated with 10 ng/mL TNF-α. (C) Translocation in response to different inducers. HUVECs were stimulated for 30 min with 10 ng/mL TNF-α, 100 ng/mL lipopolysaccharide (LPS), 10 μM phorbol myristate acetate (PMA), 10 μM prostratin, or 10 μM calcimycin (D) Nuclear translocation in response to TNF-α. HUVECs were stimulated for 30 min. The average pixel intensity was measured in both the nucleus and cytoplasm of each cell and a translocation value obtained calculating the NUC/CYT. Results are shown in boxes and whiskers. Boxes denote the interquartile range with the median represented by line inside the boxes. Whiskers show maximum and minimum values. Differences between conditions tested was assessed using the Kruskal- Wallis test. Data are a representative of two different experiments (n = 2) with at least 8 replicates per condition tested.

We next sought to determine the optimum time for NF-κB activation. HUVECs were seeded at 2500 cells/well in a 384-well plate overnight and stimulated with 10 ng/mL TNF-α. Cells were fixed at 15, 30, 45 and 60 min post cytokine stimulation, stained with NF-κB p65 antibody and Alexa Fluor 488 conjugated secondary antibody. NF-κB activation was quantified by calculating the ratio of fluorescent antibody intensity in both the nucleus and cytoplasm (NUC/CYT). As expected, little or no NF-κB was detected in nuclei of unstimulated HUVECs (1% DMSO) (Fig 2A). NF-κB was predominantly found in the cytoplasm of unstimulated cells. Stimulation of HUVECs with TNF- α resulted in NF-κB translocation from the nucleus to the cytoplasm (Fig 2A). NF-κB activation was seen as early as 15 min post stimulation and attained maximum at 30 min (Fig 2B). There was no further increase in translocation observed at 45 min and a slight decrease was observed at 60 min (Fig 2B). This same pattern was seen with different concentrations of TNF-α. Therefore, we selected 30 min as the optimal time.

Several stimuli have been identified and used to activate NF-κB [4, 24, 29]. However, cytokines and TNF-α in particular induce a more robust activation [24]. We tested the ability of different stimuli to induce NF-κB nuclear translocation (Fig 2C). HUVECs were seeded at 2500 cells/well in a 384-well plate overnight and stimulated with TNF-α, lipopolysaccharide (LPS), phorbol myristate acetate (PMA), 10 μM prostratin, or 10 μM calcimycin for 30 min. DMSO (1%) was used as the negative control. Cells were fixed and stained for NF-kβ activation. TNF-α induced the most robust NF-κB nuclear translocation (Fig 2C). There was no difference between the negative control (1% DMSO) and the other stimuli in inducing NF-κB nuclear translocation (Fig 2C). We therefore selected recombinant TNF-α as our control.

We next determined the optimal cytokine concentration for activation. HUVECs were seeded at 2500 cells/well in a 384-well plate overnight and stimulated with 5–20 ng/mL of TNF-α for 30 min. Cells were fixed, stained and imaged for NF-κB translocation. NF-κB nuclear translocation was induced with all concentrations tested (Fig 2D). There was no difference in translocation between the three concentrations (Fig 2D). To examine this further, we performed a dose response using TNF-α starting at 100 ng/mL and using a ten point, 3-fold dilution series. Although we saw no difference in NF-κB translocation between 3–100 ng/mL, less variability in response was observed with the highest concentration tested. In order to establish a robust assay with clear distinction between the negative and positive control responses, we selected 100 ng/mL of TNF-α for the rest of the experiments.

**Fig 3:**
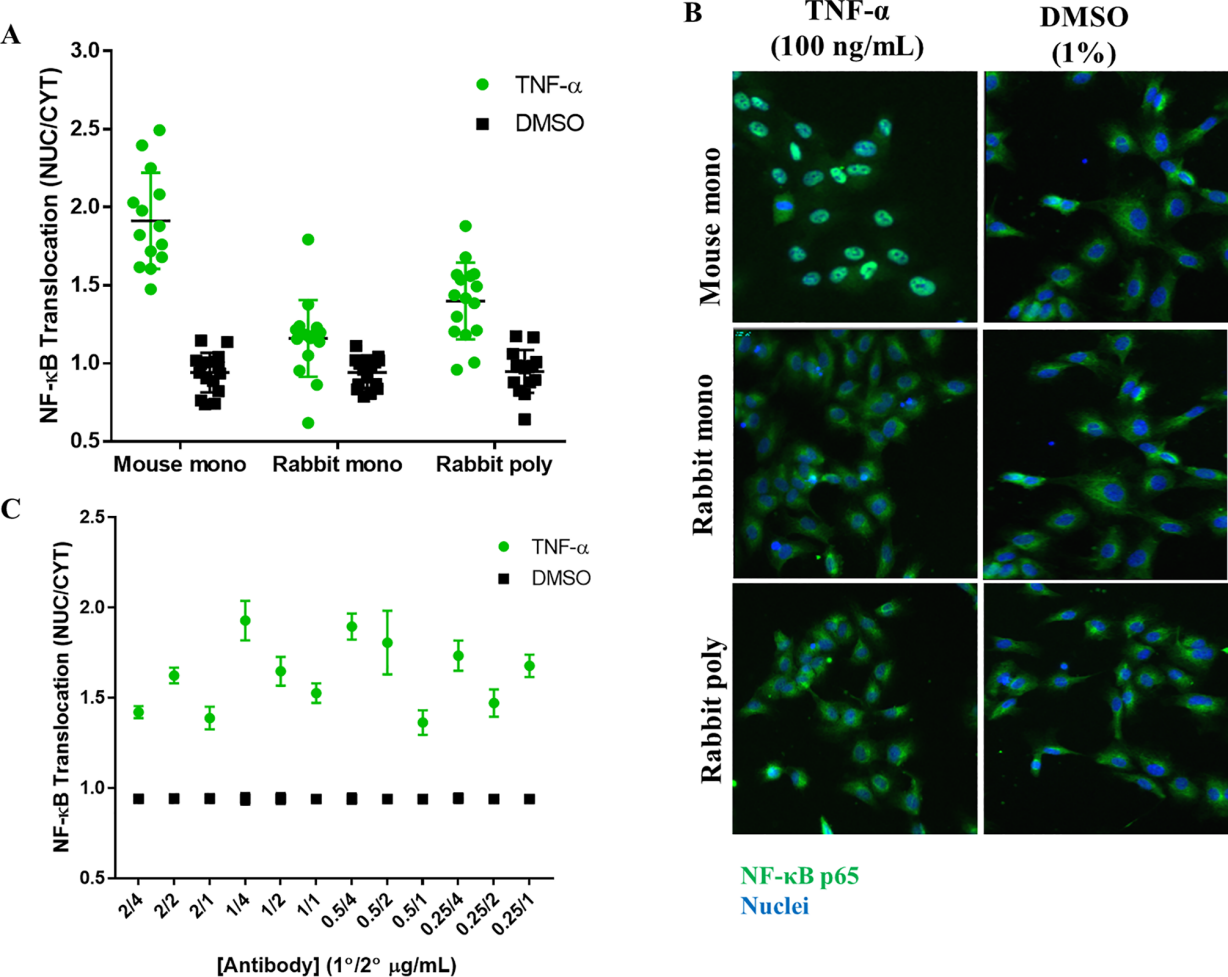
Antibody optimization. HUVECs were seeded overnight, stimulated with 100 ng/mL TNF-α and stained for NF-κB p65. NF-κB p65 average pixel intensity was measured in both the nucleus and cytoplasm of each cell and a translocation value obtained by calculating the NUC/CYT (A) Cells stained with mouse monoclonal, rabbit monoclonal and rabbit polyclonal NF-κB p65 primary antibodies. (B) Superimposed images from DAPI (nuclei) in blue and FITC (NF-κB p65) in green in TNF- α treated and DMSO control cells. Cyan stained cells are co-labelled (C) Optimization of antibody concentrations; different concentrations of primary and secondary antibodies were tested. Green circles—TNF-α stimulated cells; black squares–DMSO control. Data are a representative of two different experiments (n = 2) with at least 14 replicates per condition tested.

There can be a major variability in the specificity and performance of different antibody preparations. We determined which antibodies (primary and secondary) were optimal for our assay. For primary antibodies, we tested a rabbit monoclonal, a rabbit polyclonal and a mouse monoclonal antibody; for the secondary antibody, we tested goat anti-rabbit and goat anti-mouse. Both secondary antibody types were conjugated to Alexa Fluor 488. Our results showed consistently higher NF-κB staining with the mouse monoclonal antibody, as compared to both the rabbit monoclonal and polyclonal antibodies (Fig 3A and 3B). The mouse monoclonal antibody gave the highest NF-κB translocation signal and showed good separation between the negative and positive controls (Fig 3A). The rabbit monoclonal and polyclonal antibodies showed very little difference in staining between the positive and negative control (Fig 3A). Superimposing images from both the DAPI (nuclei, blue) and FITC (NF-κB p65, green) channels showed brightly stained cells using the mouse monoclonal antibody (Fig 3B). In addition, to higher NF-κB nuclear translocation observed with the mouse monoclonal antibody, we also observed a higher signal to background with this primary antibody compared to the rabbit monoclonal and polyclonal antibodies.

We next investigated the optimum concentrations for each primary/secondary antibody pair for the mouse monoclonal antibody and its complementary secondary antibody. Cells were stained with 0.25–2 μg/mL of mouse monoclonal antibody overnight at 4°C. For each primary antibody concentration, cells were stained with 1–4 μg/mL of goat anti-mouse secondary antibody conjugated to Alexa Fluor 488 for 1 hour at RT. We observed highest NF-κB nuclear translocation in HUVECs stained with 1 μg/mL of the primary antibody and 4 μg/mL of secondary antibody (Fig 3C). Based on above considerations, 1 μg/mL mouse monoclonal antibody and 4 μg/mL Alexa Fluor 488 goat anti-mouse antibody were selected as our assay conditions.

### Image analysis pipeline development

**Fig 4:**
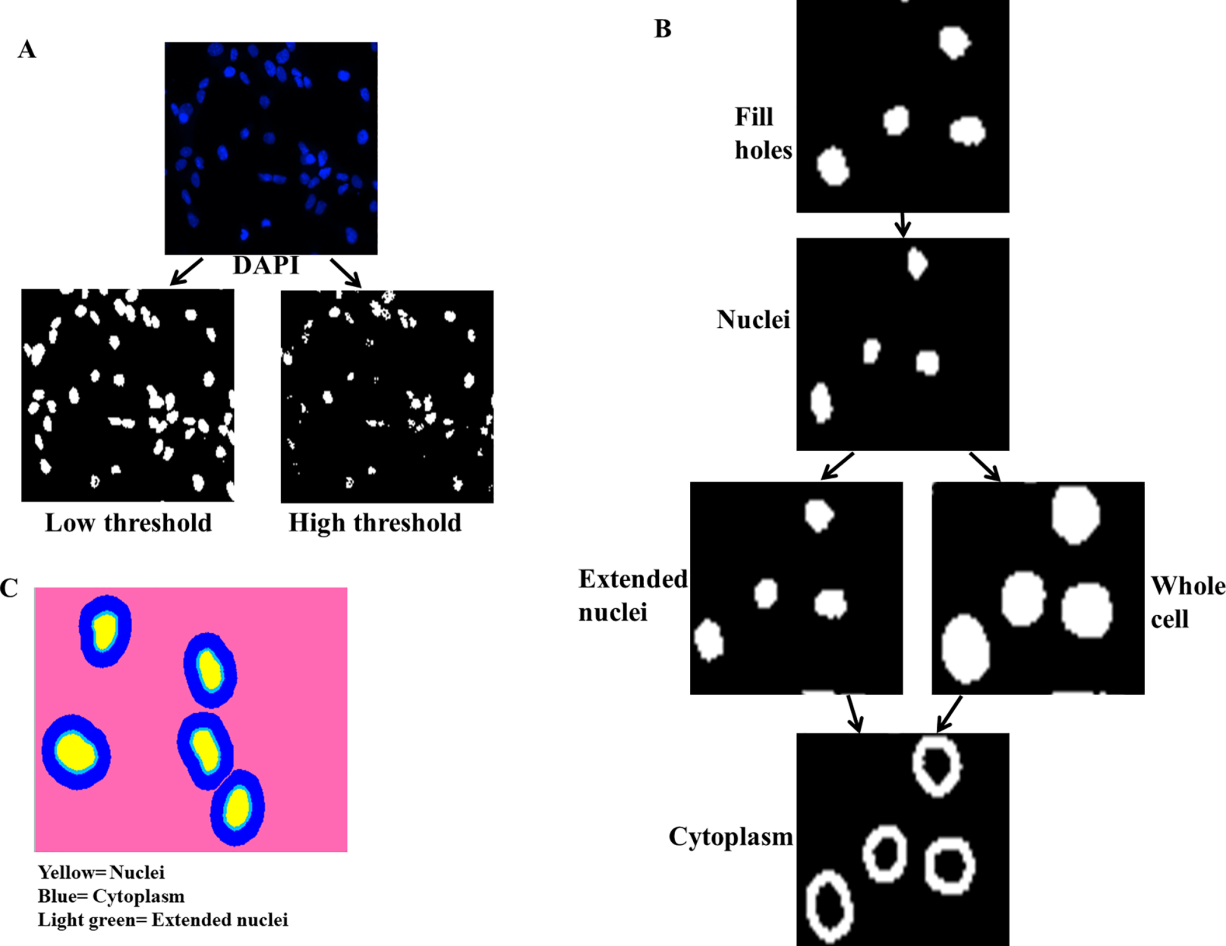
Image analysis pipeline. (A) Low and high pixel intensity threshold setting in DAPI channel to identify cells. Uneven shapes and features created with high threshold setting. Uniform features created with low threshold intensity. (B) Stepwise pipeline used to create nuclei, whole cell and cytoplasm masks. Fill holes operation done from threshold settings to create even features. Nuclei mask created from fill holes operation by shrinking features by a number of pixel intensities and size. Extended nuclei mask created from nuclei mask by increasing features by a few pixel intensities. Whole cell mask created from nuclei mask by increasing features be a number of pixel intensities. Cytoplasm mask created from both extended nuclei mask and whole cell mask (C) Different cell compartments identified using the pipeline. Yellow, blue and light green representing nuclei, cytoplasm and extended nuclei respectively.

Image acquisition and analysis were critical in accurately measuring NF-κB nuclear translocation. The custom module editor in the MetaXpress software was used to process images following acquisition. For image acquisition, we chose the 20X objective. This objective allows quantitative readouts which include numbers of cells analyzed as well as high image resolution allowing for easier identification of the nuclei than 4X objective. Higher magnification objectives had no advantage in resolution, but increased imaging acquisition time. We captured 9 fields per well in both the FITC and DAPI channels (Fig 4).

For image analysis, we set absolute values for pixel intensity in each channel to segment the features from the background. These settings were adjusted to appropriate levels for each run due to variation in experimental parameters such as exposure to light, dye and staining as well as several other factors that affected brightness in different runs. The threshold mask created from the DAPI channel was used to identify cells (Fig 4A). The threshold was set such that dim cells which were different from the background were included and very bright cells which could be an indication of cell death, cells in mitosis or just debris were excluded (Fig 4A). With a bottom threshold setting that was too high only very bright cells were captured, compared to lower and more appropriate bottom threshold settings that captured dim cells that were still significantly brighter than the background (Fig 4A). There was a lot of variation in results from analysis with high threshold settings due to the features created being uneven (Fig 4A). On the other hand, features created from low threshold settings were uniform and similar in shape (Fig 4A). For this reason, low threshold settings were used for this assay.

Following cell identification, a-5 step image analysis pipeline was created to identify the nuclei and cytoplasm by first filling holes from images from the DAPI threshold setting (Fig 4B). This was done to create even coverage across the nucleus and allowed the filled area to be included in measurements. An initial shrunk nuclei mask was created by shrinking the DAPI stained objects by a few pixels. The final nuclei mask was created from the shrunk nuclei by excluding objects based on area. Both maximum and minimum size values were used, followed by excluding objects that touched the border of the well (Fig 4B). From the final nuclei mask, two operations were performed to identify the whole cell as well as the cytoplasm mask (Fig 4B). Firstly, the nuclei mask was extended by a few pixels to create an extended nuclei mask (Fig 4B). The extended nuclei mask was created to avoid any overlap in fluorescence intensity between the nucleus and the cytoplasm. Then, to create a whole cell mask the nuclei mask was grown by a number of pixels but this time more than 3 times greater than the number used to create the extended nuclei mask. The final step in the image analysis pipeline was the creation of the cytoplasm mask by subtracting the pixels in the extended nuclei mask from the whole cell mask (Fig 4B). This image analysis pipeline enabled simple, effective and accurate identification of both the nucleus and cytoplasm of each cell and hence the measurement of NF-κB p65 antibody within each cell compartment (Fig 4C).

### Assay validation

**Fig 5:**
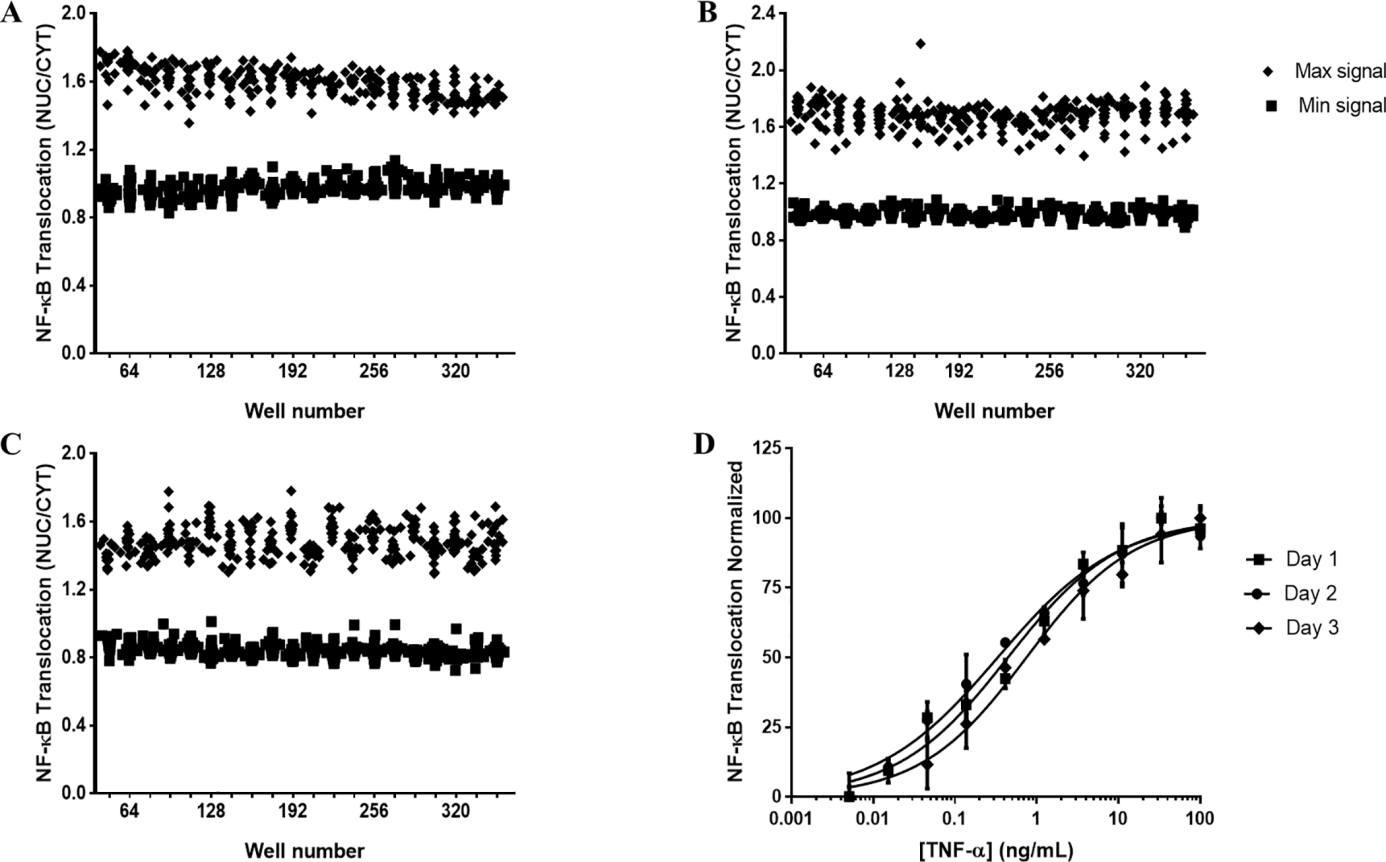
Screen validation. HUVECs were seeded overnight, stimulated with 100 ng/mL TNF-α and stained for NF-κB p65. NF-κB p65 average pixel intensity was measured in both the nucleus and cytoplasm of each cell and a translocation value obtained by calculating the NUC/CYT. Screen validation was performed as three independent experiments, each with duplicate plates of negative control (DMSO), positive control (100 ng/mL TNF-α) and dose response (10-point, 3-fold dilutions of TNF-α). (A-C) are data from 1 plate of each run for positive and negative controls. (A) Run 1 (B) Run 2 (C) Run 3. (D) Dose response curves from all three days. NUC/CYT was normalized and plotted using non- linear 4-parameter fit in order to calculate the EC_50_ = effective concentration 50%, defined as the concentration at which 50% of the maximum response is seen.

Following optimization and establishment of adequate imaging and analysis protocols, we determined whether the assay was reproducible according to NCGC guidelines for robustness testing. We determined intra-experiment and inter-experiment variability using three independent runs including negative and positive controls (Fig 5).

In each run, duplicate plates were run for full plates of maximum signal, minimum signal, and dose response. TNF-α (100ng/mL) and DMSO were used as positive and negative controls respectively. For each run the coefficient of variance (CV) was determined, as well as the Z’ of controls; for plates to pass CV had to be <20% and Z’ > 0.5. All plates met the required statistical parameters set for validation including Z’ for controls for all 3 independent experiments (Fig 5A–5C). For the dose response plates, there was little variation in EC_50_ (0.3–0.6 ng/mL) across the three independent experiments which was well within the required criteria of 3-fold variation (Fig 5D).

### High-throughput screen of small molecule libraries

**Fig 6:**
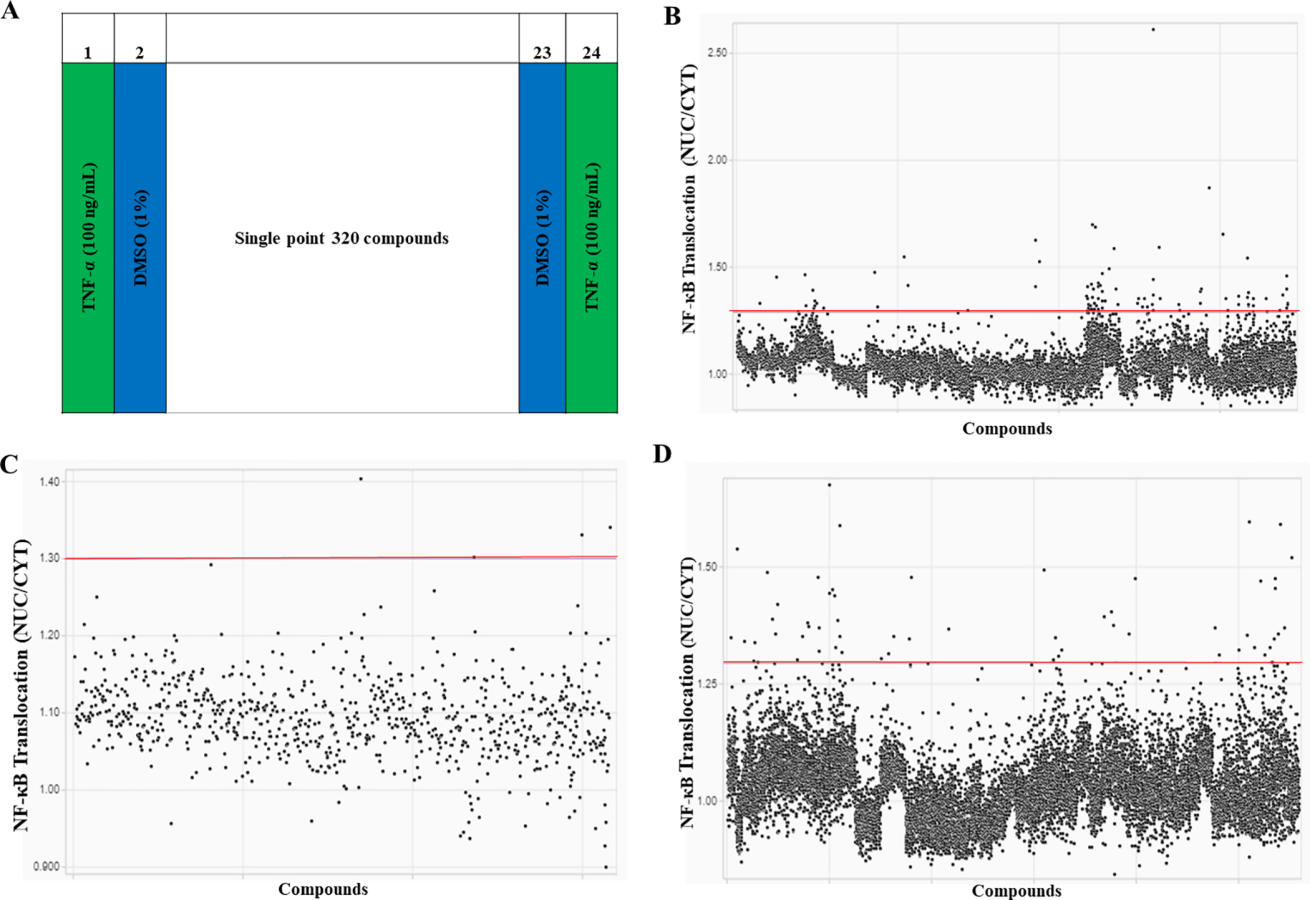
Small molecule library screen. **(**A) Plate lay out for single point compound screen at 10 μM. Positive control: 100 ng/mL TNF-α in columns 1 and 24. Negative control: 1% DMSO in columns 2 and 23. Screening data from (B) MyriaScreen II. (C) TimTec. (D) ChemBridge libraries. Red line denotes cutoff for hit identification (NUC/CYT ≥ 1.3).

We used the assay to screen 38,991 compounds from MyriaScreen II, TimTec and ChemBridge libraries. The libraries were composed of 800 natural purified products from plants, fungi and bacteria (TimTec), 10,000 compound library of small molecules (MyriaScreen II) made up of ~60% singletons with diverse structural groups (TimTec/ Sigma Aldrich) and 28,191 compounds from a 100,000 small molecule DIVERset library (ChemBridge). Each assay plate included 320 compounds screened at a fixed concentration of 10μM, plus positive and negative controls (100 ng/mL TNF-α and 1% DMSO respectively) (Fig 6A).

Compounds that induced an NF-κB nuclear translocation defined by a ratio of NUC/CYT greater than or equal to 1.3 (red line) were considered active (Fig 6B, 6C and 6D). Using these criteria, 86 compounds from MyriaScreen II (Fig 6B, Table 1), 4 compounds from TimTec (Fig 6C, Table 1) and 60 compounds from ChemBridge (Fig 6D, Table 1) induced NF-κB nuclear translocation. The hit rate was 0.9%, 0.5% and 0.2% for MyriaScreen II, TimTec and ChemBridge libraries respectively with an overall hit rate of 0.4% (Table 1).

**Table 1:** Hits from NF-κB nuclear translocation assay.

*Library*	*# compounds*	*Hits*	*Hit Rate (%)*
*MyriaScreen II*	10,000	86	0.9
*TimTec*	800	4	0.5
*ChemBridge*	28,191	60	0.2
*Total*	38,891	150	0.4

### Confirmation and validation of identified hits

**Fig 7:**
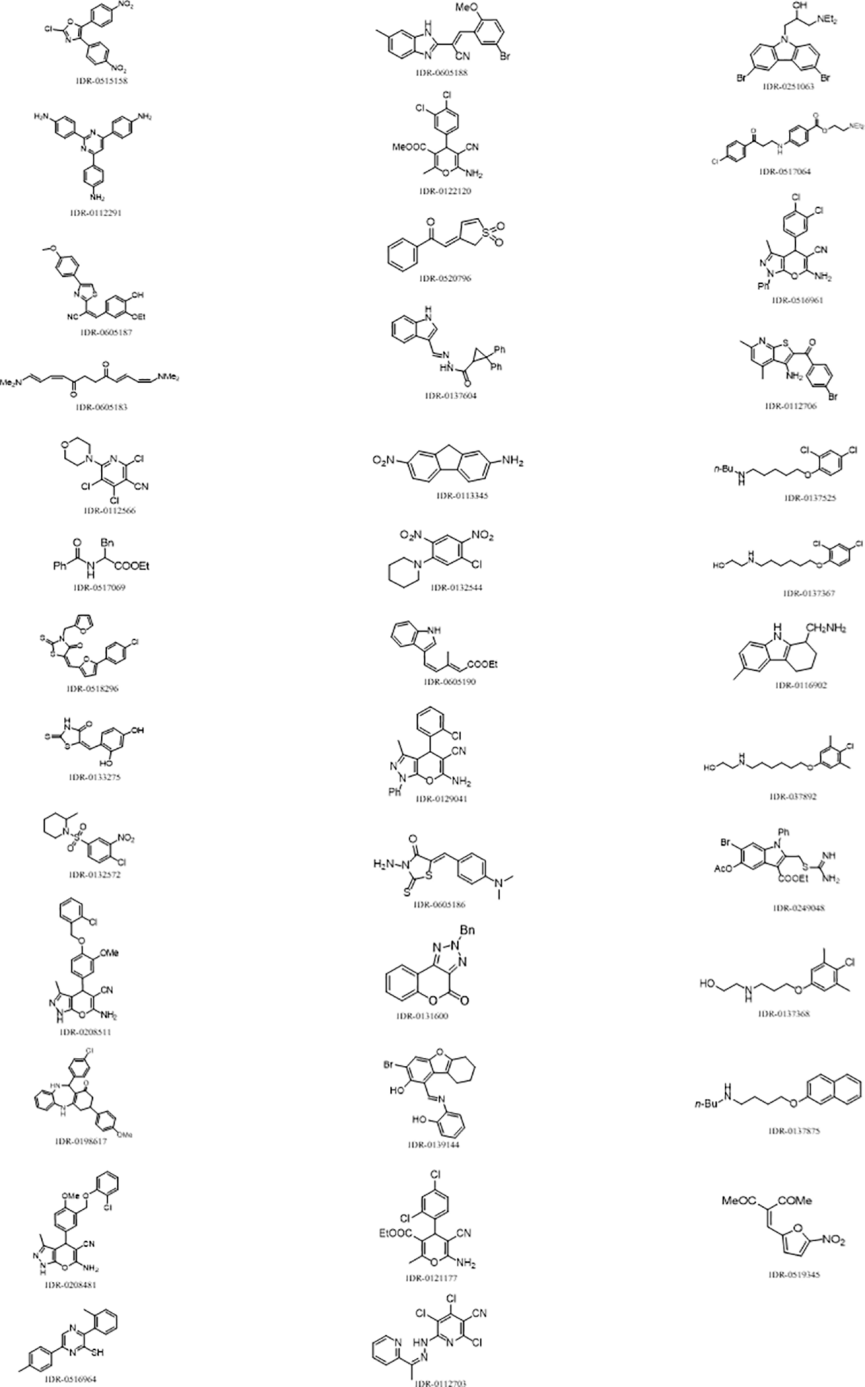
Confirmed hits and structures: Small molecules confirmed as hits in dose-response experiment with their corresponding structures.

**Fig 8:**
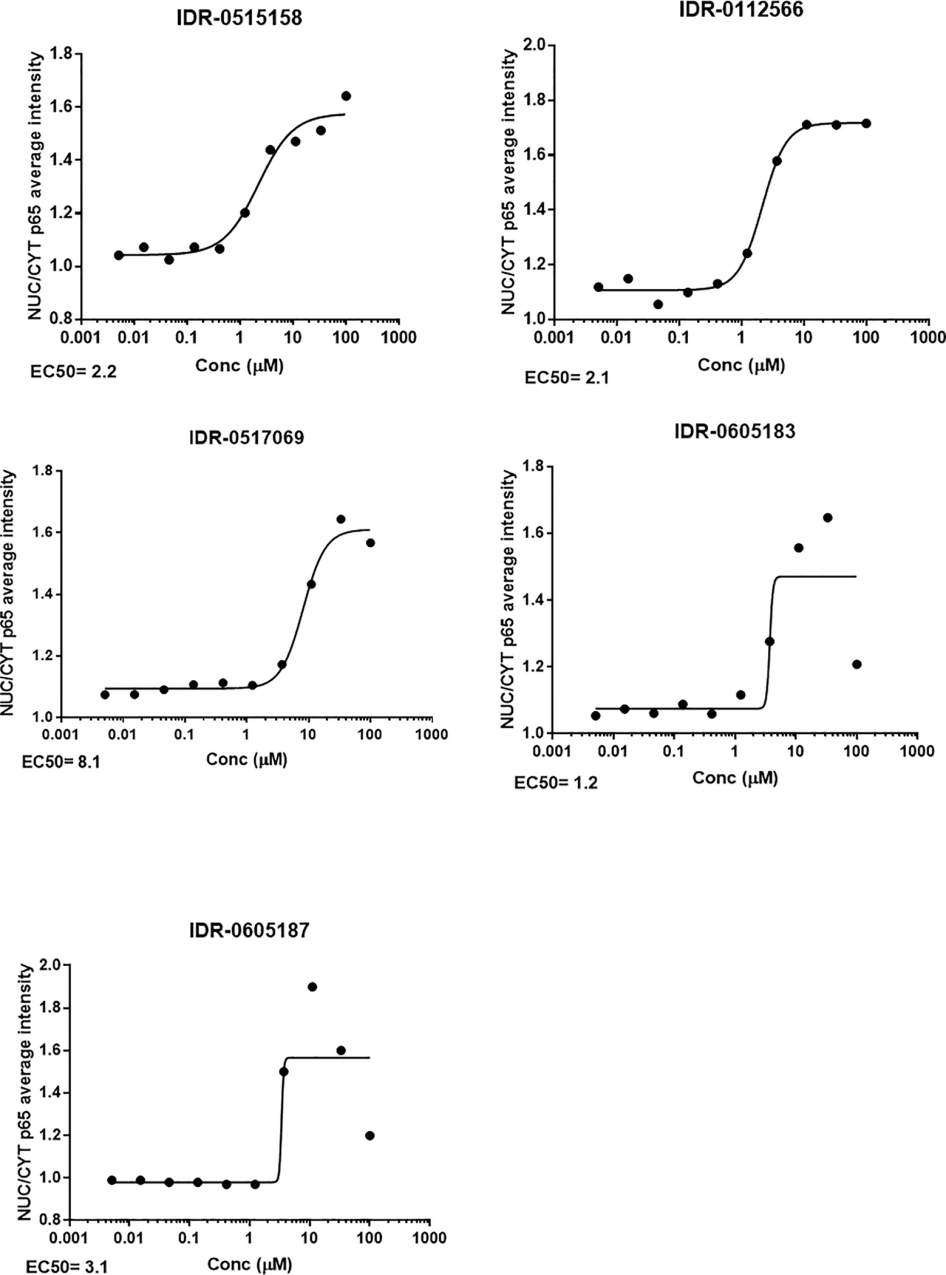
Dose response curves for compounds with good selectivity. HUVECs were seeded at 2500 cells/well in 384 well plates overnight. A dose response (10-point, 3-fold dilutions) of compounds starting at 100μM was done, plates stained, imaged and NF-κB nuclear translocation (NUC/CYT) determined for each concentration of each compound. Curve was fitted using a non- linear 4-parameter fit on Graphpad Prism 7.04 and the EC_50_ for each compound determined. Data are a representative of three different experiments (n = 3).

Following identification of hits from the primary screen, we confirmed the activity of the compounds to induce NF-κB nuclear translocation. Of the 150 compounds, we selected 103 for further study. The remainder were excluded for reasons such as known cytotoxic properties or instability. All 103 hits were re-ordered from various vendors and tested as a dose response using a 10-point, 3-fold serial dilution, with a starting concentration of 100 μM. For each compound, we calculated the EC_50_. From our primary screen, 38 compounds were active in a dose response assay (Fig 7). Activity was defined as compounds having an EC_50_ <50 μM. Of these hits, 22 reconfirmed as active on retest in triplicate (Table 2 and Fig 8).

**Table 2:** Activity for confirmed hits. EC_50_ = Activity in HUVECs, IC_50_ = Cytotoxicity in HepG2, D = Unique compound identification number. Data are mean ± SD of three independent experiments for hit confirmation (for * compounds only N = 2 was run) and two independent experiments for cytotoxicity. The selectivity index (SI) was determined as IC_50_/EC_50_.

ID	EC50	IC50	SI
IDR-0112566	3.8 ± 1.4	28 ± 0.7	7.4
IDR-0112703	6.3 ± 6.0	13 ± 5.0	2.1
IDR-0112706	13 ± 4.5	27 ± 3.5	2.1
IDR-0113345	8.6 ± 8.9	54 ± 5.0	6.3
IDR-0122120	6.6 ± 8.1*	40 ± 5.0	6.1
IDR-0131600	17 ± 3.7	32 ± 0.7	1.9
IDR-0133275	18 ± 1.1*	>100	>5.5
IDR-0137368	17 ± 14	19 ± 4.9	1.1
IDR-0137875	15 ± 3	16 ± 0.71	1.1
IDR-0137892	9.8 ± 3	7.3 ± 1.8	0.7
IDR-0139144	12 ± 10	18 ± 4.2	1.5
IDR-0208481	13 ± 5*	41 ± 1.4	3.2
IDR-0515158	2.6 ± 0.53*	86 ± 17	33
IDR-0516961	25 ± 17	46 ± 18	1.8
IDR-0516964	19 ± 14	78 ± 32	4.1
IDR-0517069	10 ± 1.6	>100	>10
IDR-0518296	2.4 ± 1.4	17 ± 12	7.1
IDR-0520796	5.0 ± 5.6	6.6 ± 1.6	1.3
IDR-0605183	5.0 ± 6.0	31 ± 3.5	6.2
IDR-0605186	15 ± 5.9	>50	>3.3
IDR-0605187	5.9 ± 3.4	51 ± 31	8.6
IDR-0605188	4.7 ± 3.8	17 ± 13	3.6

We next assessed our hit compounds for cytotoxicity in a human hepatocyte cell line. The majority of the compounds had some effect on HepG2 (Table 2). The selectivity index (SI) for each compound was determined as IC_50_/EC_50_. Ten of the compounds showed good selectivity (SI>5) (Table 2), six had some selectivity (SI of 2–5) and seven had no selectivity (SI <2) (Table 2). The compounds with SI >2 show some promise for further investigation.

## Discussion

In this study, we describe the validation and implementation of a screen for compounds with the ability to activate the NF-κB pathway. The principle of the assay is to measure the translocation of NF-κB from the cytoplasm (where it is predominant in an inactive state in resting cells) to the nucleus following stimulation by microscopy. HUVECs were chosen for the assay development and primary screen as opposed to an immune cell, since they are relative easy to culture, they are primary cells and they have large nucleus and cytoplasm. A clear distinction between the nucleus and cytoplasm is important for this assay to allow accurate determination of translocation. Thus cells with relatively large cytoplasmic to nuclear area ratio are recommended [4]. We excluded the use of human macrophage-like cell lines (THP-1), since they require differentiation induced by PMA, which itself can act as an NF-κB inducer [29, 30]. However, primary immune cells and cell lines can be used for low throughput and secondary experiments to confirm hits from primary screen with the same approach detailed above applied with some modifications [4].

Two pathways are involved in NF-κB signaling, the canonical/classical and the non-canonical/alternative pathways [31, 32]. For this screen, we were interested in the canonical pathway of NF-κB signaling since its activation results in immune response and inflammatory gene expression [33, 34]. The primary activators of this pathway are pro-inflammatory signals such as cytokines, pathogen-associated molecular patterns (PAMPs), and some danger-associated molecular patterns (DAMPs) [33]. Among these, TNF-α has been shown to be the most robust activator of NF-κB [15, 24]. We also observed the same result when we investigated different activators of NF-κB in our assay. This cytokine worked well as a positive control for our primary screen as it had previously for HeLa cells [24].

IκB can take up to an hour to accumulate in the cytoplasm after NF-κB activation and translocation, [8, 9]. When this happens, IκB may translocate to the nucleus and bind to NF-κB inhibiting its activity as well as diminishing its signal [10]. Establishing an optimal activation time was therefore very critical for this assay. We observed optimal NF-κB activation at 30–45 min post stimulation of HUVECs with TNF-α consistent with previous work.

We screened ~ 40,000 compounds for NF-κB nuclear translocation. We had a hit rate of 0.38%, similar to other similar screens [35, 36]. Hit selection was a critical step in the screening process. Two main strategies have often been used to identify hits: (i) to test if the compounds have a strong enough effect to reach a set level [37–39] and (ii) to rank the compounds by magnitude of effect followed by selection of the largest number practical for confirmation and validation [40, 41]. For our hit selection, we used the second strategy whereby compounds were ranked by NF-κB p65 NUC/CYT. Our confirmation rate was <40%, which is lower than we normally see for whole cell screens. There are several reasons why compounds may not confirm, including the fact that many of our compounds were resupplied by alternative vendors meaning that possible changes in purity or contaminants could arise. Nevertheless, the confirmation rate is still acceptable and we have a number of promising compounds to pursue.

### Conclusions

We have successfully optimized and validated an NF-κB nuclear translocation assay with robustness and reproducibility for high content, high-throughput screening for HUVECs. Using this screen, we identified small molecule- activators of NF-κB. Further studies should focus on characterizing these molecules *in vitro* with human mixed cell populations as well as in immune cell lines and *in vivo* for adjuvant properties.
